# Particulate matter 2.5 damages skin cells by inducing oxidative stress, subcellular organelle dysfunction, and apoptosis

**DOI:** 10.1007/s00204-018-2197-9

**Published:** 2018-03-26

**Authors:** Mei Jing Piao, Mee Jung Ahn, Kyoung Ah Kang, Yea Seong Ryu, Yu Jae Hyun, Kristina Shilnikova, Ao Xuan Zhen, Jin Woo Jeong, Yung Hyun Choi, Hee Kyoung Kang, Young Sang Koh, Jin Won Hyun

**Affiliations:** 1 Jeju National University School of Medicine and Jeju Research Center for Natural Medicine, Jeju, 63243 Republic of Korea; 2 Laboratory of Veterinary Anatomy, College of Veterinary Medicine, Jeju National University, Jeju, 63243 Republic of Korea; 3 Department of Biochemistry, College of Oriental Medicine, Dongeui University, Busan, 47340 Republic of Korea

**Keywords:** PM_2.5_, Oxidative stress, Apoptosis, Endoplasmic reticulum stress, Mitochondrial damage, Autophagy

## Abstract

The skin is the largest organ of the human body and the one mostly exposed to outdoor contaminants. To evaluate the biological mechanisms underlying skin damage caused by fine particulate matter (PM_2.5_), we analyzed the effects of PM_2.5_ on cultured human keratinocytes and the skin of experimental animals. PM_2.5_ was applied to human HaCaT keratinocytes at 50 µg/mL for 24 h and to mouse skin at 100 µg/mL for 7 days. The results indicate that PM_2.5_ induced oxidative stress by generating reactive oxygen species both in vitro and in vivo, which led to DNA damage, lipid peroxidation, and protein carbonylation. As a result, PM_2.5_ induced endoplasmic reticulum stress, mitochondrial swelling, and autophagy, and caused apoptosis in HaCaT cells and mouse skin tissue. The PM_2.5_-induced cell damage was attenuated by antioxidant *N*-acetyl cysteine, confirming that PM_2.5_ cellular toxicity was due to oxidative stress. These findings contribute to understanding of the pathophysiological mechanisms triggered in the skin by PM_2.5_, among which oxidative stress may play a major role.

## Introduction

Global air pollution has become a major threat to human health. This worldwide problem is especially relevant to the release of fine particulate matter (PM_2.5_), which has the aerodynamic diameter less than 2.5 µm and originates from incomplete coal combustion and diesel vehicle exhaust in Korea (Lee et al. 2005; Jung et al. 2017). In recent years, the relationship between PM_2.5_ production and public health hazards has attracted an increasing attention. Several toxicological and epidemiological studies have suggested that PM_2.5_ exerts negative biological effects on several major organs, including the lung (Liu et al. 2017), immune system (Zhao et al. 2013), cardiovascular system (Du et al. 2016), and nervous system (Wang et al. 2017). Among the affected organs, the skin is the primary tissue exposed to ambient pollutants and, similar to the respiratory tract, presents an interface between the body and surrounding atmosphere.

PM_2.5_-carrying organic chemicals such as polycyclic aromatic hydrocarbons (PAHs) are highly lipophilic and easily penetrate the skin (Krutmann et al. 2014). PAHs are potent activators of the aryl hydrocarbon receptor (AhR), which is a ligand-dependent transcription factor expressed in keratinocytes and melanocytes (Fritsche et al. 2007; Jux et al. 2011). AhR activation by PAHs upregulates the expression of cytochrome P450 (CYP1A1) involved in the metabolism of xenobiotics (Vogel et al. 2016) and promotes generation of intracellular reactive oxygen species (ROS) (Costa et al. 2010). Accumulated evidence indicates that oxidative stress is a common mechanism of PM_2.5_-induced damage (Gualtieri et al. 2012; Kouassi et al. 2010). Recently, the effect of PM_2.5_ on the skin has attracted attention of both clinical dermatologists and basic scientists (Han et al. 2016; Li et al. 2017), who recognized ambient PM_2.5_ as a crucial risk factor in skin diseases. Thus, PM_2.5_ was shown to aggravate symptoms in children with allergic dermatitis and eczema (Song et al. 2011), and to promote inflammatory disorders, aging, androgenetic alopecia, and cancers of the skin (Kim et al. 2016).

Mitochondria are unique double-membrane subcellular organelles that provide energy through oxidative phosphorylation and participate in metabolic and genetic processes in the body. Once mitochondria are disrupted, their dysfunction leads to reduced generation of ATP and higher production of ROS. Mitochondria are targeted by environmental pollutants such as PM_2.5_ (Guo et al. 2017) and mitochondrial damage may be a critical part of the pathophysiological mechanisms induced by PM_2.5_ exposure. Oxidative stress has been shown to be an initiator and major contributor to both endoplasmic reticulum (ER) stress (Hotamisligil 2010; Kaneto et al. 2005) and lysosome-mediated autophagy (Lee et al. 2012); however, the research on molecular pathways linking atmospheric PM_2.5_ and skin damage is limited. Although skin is the organ mostly exposed to PM_2.5_, the association of skin-damaging effects of PM_2.5_ with oxidative stress and dysfunction of subcellular organelles such as mitochondria, ER, and lysosomes is still not fully elucidated. In this study, we investigated the effects of PM_2.5_ on the induction of oxidative stress and structure of subcellular organelles using in vitro and in vivo models and explored the mechanisms underlying PM_2.5_ toxicity for the skin.

## Materials and methods

### Preparation of PM_2.5_

Diesel particulate matter NIST 1650b (PM_2.5_) was purchased from Sigma-Aldrich, Inc. (St. Louis, MO, USA). PM_2.5_ stock solution (25 mg/mL) was prepared in dimethyl sulfoxide (DMSO) and sonicated for 30 min to avoid agglomeration of the suspended PM_2.5_ particles. Experiments were performed within 1 h of stock preparation to avoid variability in PM_2.5_ composition in solution.

### Cell culture

Human HaCaT keratinocytes (Amore Pacific Company, Gyeonggi-do, Republic of Korea) were maintained at 37 °C in an incubator with a humidified atmosphere of 5% CO_2_. Cells were cultured in Dulbecco-modified Eagle medium (DMEM) containing 10% heat-inactivated fetal bovine serum and antibiotic–antimycotic (100 units/mL penicillin, 100 µg/mL streptomycin, and 0.25 µg/mL amphotericin B) (Gibco, Life Technologies Co., Grand Island, NY, USA).

### Animal experiment

In vivo experiments were conducted using HR-1 hairless male mice (OrientBio, Kyungki-do, Republic of Korea) in accordance with the guidelines for the care and use of laboratory animals at Jeju National University (Jeju, Republic of Korea) (permit number: 2017-0026). Mice were randomly divided into three groups (*n* = 4 each): control, and treated with PM_2.5_ or *N*-acetyl cysteine (NAC; Sigma-Aldrich) + PM_2.5_. PM_2.5_ was dispersed in propylene glycol to the concentration of 100 µg/mL, spread on a nonwoven polyethylene pad over a 1 cm^2^ area, and applied to the dorsal skin of mice for 7 consecutive days. At the end of the treatment, the exposed skin tissue was immediately dissected for histological and biochemical analysis as previously described (Lee et al. 2016).

### ROS measurement

Cells were incubated with different concentrations of PM_2.5_ (25, 50, 75, and 100 µg/mL) for 24 h or treated with PM_2.5_ at a concentration of 50 µg/mL for different times (1, 2, 4, 8, 12, and 24 h). After staining with 25 µM 2′,7′-dichlorodihydrofluorescein diacetate (H_2_DCFDA; Molecular Probes, Eugene, OR, USA) dye for 10 min, H_2_DCF fluorescence was detected by flow cytometry (Becton Dickinson, Mountain View, CA, USA) and analyzed using the Cell Quest software. For imaging, cells were plated in a 4-well glass chamber slide, treated with 1 mM NAC and/or 50 µg/mL PM_2.5_, and analyzed for intracellular and mitochondrial ROS production after staining with H_2_DCFDA and dihydrorhodamin 123 (DHR 123; Molecular Probes), respectively, for 30 min. Images of stained cells were generated by confocal microscopy as previously described (Kim and Yoo 2016; Soeur et al. 2017). To detect ROS in zebrafish treated with NAC and/or PM_2.5_, they were incubated with 10 µM H_2_DCFDA for 30 min in the dark at 28.5 °C. After anesthesia with 1-phenoxy-2-propanol (1/500 dilution; Acros Organics, Morris Plains, NJ, USA), the stained zebrafish were observed under a fluorescence microscope (Zeiss AX10, Carl Zeiss, Göttingen, Germany) (Jeong et al. 2017).

### Hoechst 33342/propidium iodide (PI) staining

Cells were treated with different concentrations of PM_2.5_ (25, 50, 75, and 100 µg/mL) for 24 h or pre-treated with 1 mM NAC for 1 h and then treated with 50 µg/mL of PM_2.5_ for 24 h and then stained with DNA-specific fluorescent dyes Hoechst 33342 (10 µM) and propidium iodide (PI; 5 µg/mL) (both from Sigma-Aldrich). Cells with fragmented nuclei stained with Hoechst 33342 were considered apoptotic and those stained with PI were considered necrotic. Cell staining was visualized under a fluorescence microscope equipped with a CoolSNAP-Pro color digital camera (Media Cybernetics, Rockville, MD, USA) and the proportions of apoptotic and neurotic cell were quantified.

### Trypan blue assay

Cells were seeded in 35-mm culture dishes at a concentration of 1.0 × 10^5^ per mL, cultured for 16 h, and treated with different concentrations of PM_2.5_ (25, 50, 75, and 100 µg/mL) for 24 h. Then, 5 µL of 0.1% trypan blue solution was added to 0.1 mL cell suspension for 5 min at room temperature, and the numbers of viable and dead cells were determined under a microscope using 10× magnification. Cell viability (%) was calculated as: unstained cells/(unstained cells + stained cells) × 100%.

### Detection of 8-oxoguanine

Cellular DNA was isolated using the G-DEX™ IIc Genomic DNA Extraction Kit (iNtRON Biotechnology, Inc., Sungnam, Kyungki-Do, Republic of Korea) and quantified by spectrophotometry. The amount of 8-hydroxy-2-deoxyguanosine (8-OHdG, the deoxyriboside form of 8-oxoG) in DNA was determined using the BIOXYTECH^®^ 8-OHdG-EIA™ kit (OXIS Health Products, Inc., Portland, OR, USA) according to the manufacturer’s instructions. The amount of 8-oxoG was also estimated by a fluorescence-binding assay: cells were fixed and permeabilized with ice-cold methanol for 15 min and 8-oxoG was visualized with avidin-TRITC conjugate (Sigma-Aldrich) under a confocal microscope (Piao et al. 2011).

### Single cell gel electrophoresis (Comet assay)

Cell-coated slides were immersed in lysis buffer (2.5 M NaCl, 100 mM Na-EDTA, 10 mM Tris, 1% Trion X-100, and 10% DMSO, pH 10) for 1 h at 4 °C, subjected to electrophoresis, stained with ethidium bromide, and observed under a fluorescence microscope equipped with an image analysis software (Kinetic Imaging, Komet 5.5, UK) as previously described (Park et al. 2017). The percentage of the total fluorescence in the comet tail and the length of the tail was recorded in 50 cells per slide.

### Lipid peroxidation assay

Cells were stained with 5 µM of a fluorescent probe diphenyl-1-pyrenylphosphine (DPPP; Molecular Probes) as described (Morita et al. 2016) and analyzed using an Olympus FV1200 laser scanning microscope equipped with the FV10-ASW viewer 4.2 software. Mouse skin tissue was analyzed by immunohistochemistry using an antibody to 4-hydroxy-2-nonenal (4-HNE) (Cosmo Bio Co., Tokyo, Japan).

### Protein carbonylation

Total cellular proteins were extracted with lysis buffer and quantified by spectrophotometry, and protein carbonylation was determined using an OxiSelect™ protein carbonyl ELISA kit (Cell Biolabs, San Diego, CA, USA) according to the manufacturer’s instructions. In tissues, protein carbonylation was assessed using an immunohistochemical staining kit (Cosmo Bio Co.)

### Western blotting

Cell and mouse skin lysates were subjected to SDS-PAGE, and the separated proteins were transferred to membranes and incubated with primary antibodies against phospho-H2A.X (Ser139), CHOP, phospho-PERK, beclin-1, LC3B, caspase-3, and caspase-9 (Cell Signaling Technology, Beverly, MA, USA), GRP78, Bax (Santa Cruz Biotechnology, Santa Cruz, CA, USA), phospho-IRE1 (Abcam, Cambridge, MA, USA), and actin (Sigma-Aldrich) followed by the incubation with a secondary antibody (Pierce, Rockford, IL, USA). Protein bands were detected using an Amersham Enhanced Chemiluminescence Plus Western Blotting Detection system (GE Healthcare Life Sciences, Buckinghamshire, UK).

### ER staining

Cells were reacted with an ER-tracker blue-white DPX dye (Molecular Probes) for 30 min, and images were taken under a confocal microscope (Li et al. 2015).

### Quantification of Ca^2+^ level

Cells were loaded for 30 min with 10 µM fluo-4-acetoxymethyl ester (Fluo-4-AM) or with Rhod-2 acetoxymethyl ester (Rhod-2 AM) (Molecular Probes) to detect intracellular and mitochondrial Ca^2+^, respectively, and fluorescence was measured by confocal microscopy (Wang et al. 2016).

### Mitochondrial membrane potential (Δ_ψm_) measurement

Mitochondrial Δ_ψ_ was analyzed by confocal microscopy after staining with 5,5′,6,6′-tetrachloro-1,1′,3,3′-tetraethylbenzimidazolylcarbocyanine iodide (JC-1, Invitrogen, Carlsbad, CA, USA), a lipophilic cationic fluorescence dye (Yao et al. 2016).

### Acridine orange staining

To analyze autophagy, cells were reacted with acridine orange (Invitrogen) for 15 min and fluorescence was measured using a fluorescence microscope (BH2-RFL-T3; Olympus, Tokyo, Japan) (Farah et al. 2016). Depending on the acidity, autophagic lysosomes appeared as orange/red fluorescent cytoplasmic vesicles, while the nuclei were stained green.

### LC3 transfection and detection of punctate LC3-positive structures

Membrane-bound microtubule-associated protein 1 light chain 3 (LC3) is present in the autophagic double-membrane structure, which is an important marker of autophagy (Fazeli and Wehman 2017). Cells were transfected with GFP-tagged LC3 using Lipofectamine reagent (Invitrogen) according to the manufacturer’s instructions and GFP-LC3 fluorescence was observed under a confocal microscope.

### Histological analysis

Skin pieces were fixed in 4% paraformaldehyde, embedded in paraffin, and cut into 5 µm sections, which were then deparaffinized and stained with hematoxylin and eosin. The height of epidermis (from the stratum basale to the stratum corneum) was measured in ten randomly chosen fields from three representative sections per group by microscopy at 100× magnification using a digital camera. Immunohistochemistry was performed by incubating skin sections with primary anti-Bax antibody (1:400; Abcam, Cambridge, MA, USA) for 1 h and the reaction was visualized using an ABC Elite kit (Vector Labs, Burlingame, CA, USA). The sections were counterstained with hematoxylin before mounting. For the in situ detection of apoptotic cells in skin sections, the DeadEnd colorimetric TUNEL system (Promega, Wisconsin, WI, USA) was used according to the manufacturer’s recommendation.

### Transmission electron microscopy (TEM)

Cells and tissues were fixed, dehydrated, incubated with increasing concentrations of propylene oxide dissolved in ethanol, and infiltrated with increasing concentrations of Eponate 812 resin. Samples were baked in a 65 °C oven overnight, sectioned in an ultramicrotome, and examined by TEM using a field electron emission unit (JEM-2100F, JEOL) at the Korean Basic Science Institute (Chuncheon, Republic of Korea).

### Statistical analysis

Statistical significance of the difference between groups was determined by analysis of variance and Tukey’s tests using the SigmaStat version 3.5 software (Systat Software Inc., San Jose, CA, USA). All data are presented as the mean ± standard error. *p* < 0.05 was considered to indicate statistically significant difference.

## Results

### PM_2.5_ induced oxidative stress both in vitro and in vivo

**Fig. 1:**
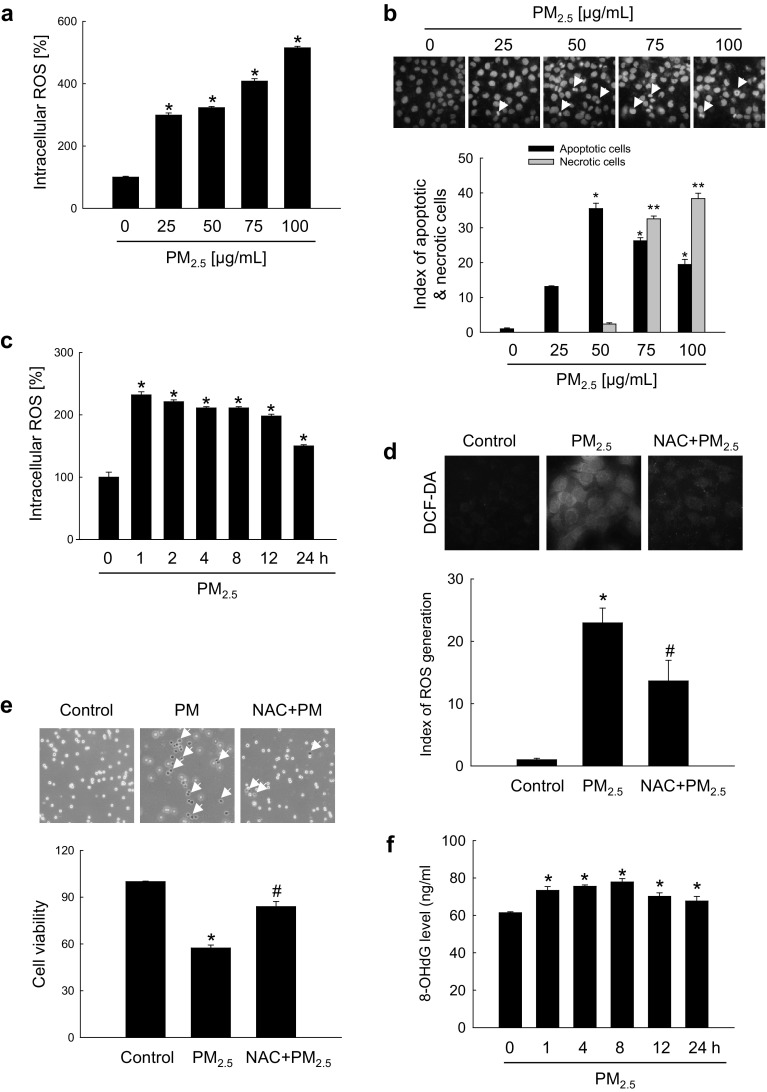

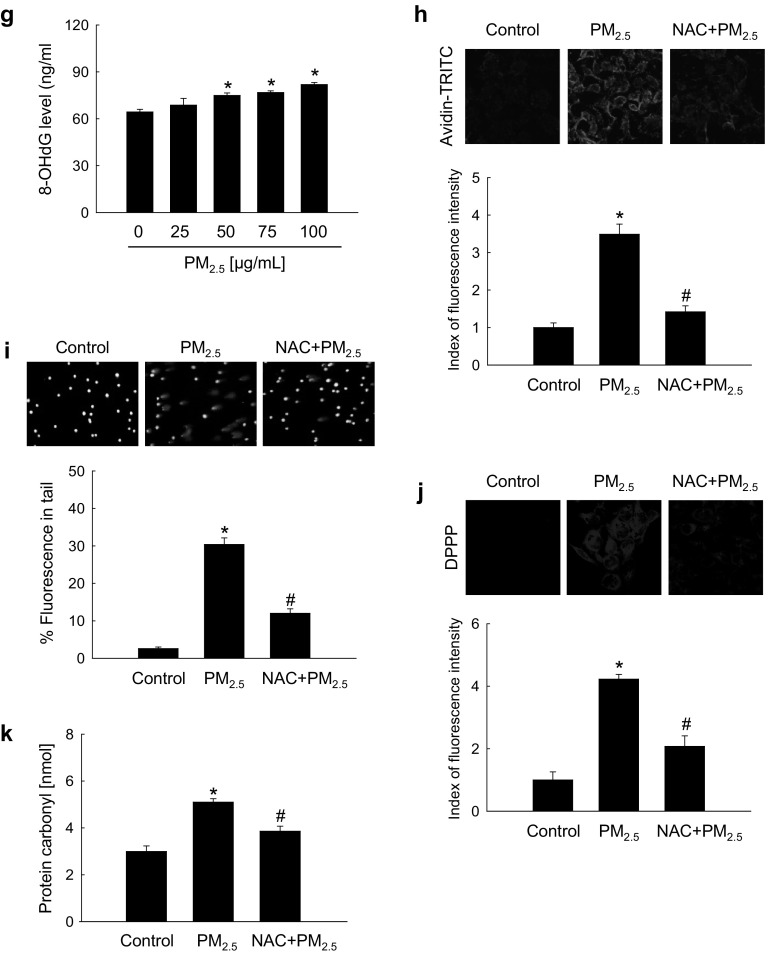

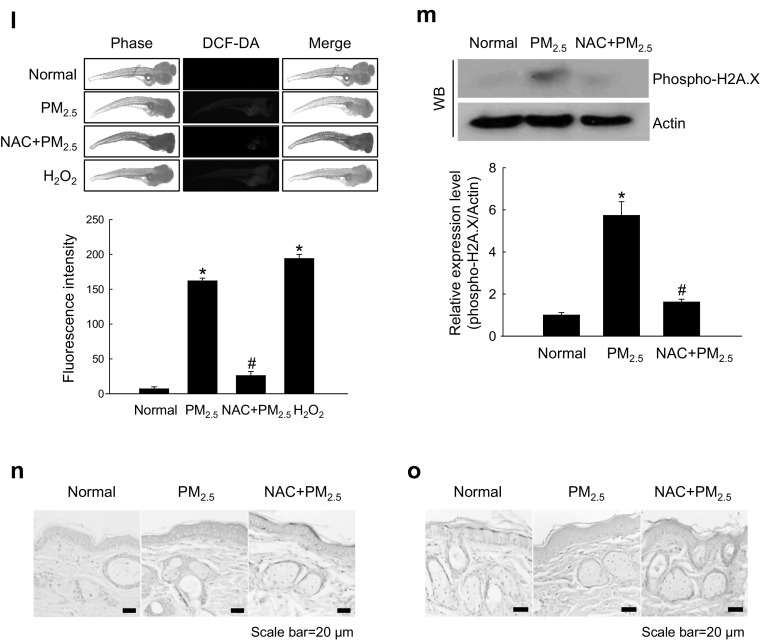
PM_2.5_ induces ROS production leading to oxidative damage. Cells were treated with PM_2.5_ at the indicated concentrations for 24 h. **a** ROS generation was assessed by the H_2_DCFDA assay. **b** Apoptotic and necrotic cells were detected by Hoechst 33342 and PI nuclear staining, respectively. Arrows indicate apoptotic bodies and red color indicates necrotic cells. **c** Cells were treated with 50 µg/mL PM_2.5_ for the indicated times and ROS generation was assessed by the H_2_DCFDA assay. Cells were pre-treated with NAC (1 mM) for 1 h and then treated with PM_2.5_ (50 µg/mL) for 24 h. **d** ROS levels were assessed by confocal microscopy after H_2_DCFDA staining. **e** Cell viability was analyzed by trypan blue exclusion. Cells were treated with 50 µg/mL PM_2.5_ **f** for the indicated times or **g** with the indicated concentrations of PM_2.5_ for 8 h and analyzed for the generation of 8-oxoG in DNA by avidin-TRITC binding using confocal microscopy. **i** DNA damage was evaluated by the Comet assay; representative images show comet tails and the graph shows quantification of cellular DNA damage. **j** Lipid peroxidation was analyzed by confocal microscopy after DPPP staining. **k** Protein oxidation was assayed by measuring carbonyl formation. **l** Zebrafish was pre-treated or not with NAC and then treated with PM_2.5_ (50 µg/mL) for 24 h, and analyzed for ROS production by H_2_DCFDA staining; zebrafish treated with H_2_O_2_ was used as a positive control. Mouse dorsal skin was treated with PM_2.5_ (100 µg/mL) for 7 days. **m** Tissue lysates were analyzed for H2A.X expression by immunoblotting; actin was used as loading control. **n** Immunohistochemistry of mouse tissue to analyze 4-HNE levels as a marker of lipid peroxidation; signals were detected in a peroxidase reaction (brown), and slides were counterstained with hematoxylin; magnification: ×400. **o** Protein carbonylation in mouse tissue was assessed using an immunohistochemical staining kit for protein carbonyls. *p < 0.05 compared to control groups and ^#^*p* < 0.05 compared to PM_2.5_-treated groups. (Color figure online)

To investigate the potential role of oxidative stress induced by PM_2.5_, we measured ROS generation and cellular damage in PM_2.5_-treated human keratinocytes and mouse skin. Figure 1a shows that PM_2.5_ treatment promoted the production of ROS in a dose-dependent manner as evidenced by H_2_DCFDA staining. Analysis of Hoechst 33342/PI staining indicated that PM_2.5_ at a concentration of 50 µg/mL induced apoptosis (Hoechst 33342-stained cells); however, at concentrations above 75 µg/mL, PM_2.5_ induced necrosis (PI-stained cells) (Fig. 1b). We used 50 µg/mL PM_2.5_ as the optimal concentration in further experiments. ROS generation was increased starting from 1 h up to 24 h of treatment with 50 µg/mL PM_2.5_ (Fig. 1c). Next, we determined whether PM_2.5_ induced cytotoxicity via ROS generation. An antioxidant NAC was not toxic for HaCaT cells at concentrations up to 1 mM (MTT test; data not shown); therefore, 1 mM NAC was used in this study. Confocal microscopy indicated that green fluorescence was increased in PM_2.5_-treated cells compared to control, but the effect was suppressed by 1 mM NAC (Fig. 1d), indicating that PM_2.5_ stimulated ROS production in keratinocytes. Furthermore, PM_2.5_ induced cytotoxicity as evidenced by trypan blue exclusion; however, 1 mM NAC reduced it (Fig. 1e), suggesting that PM_2.5_ caused cytotoxicity via ROS. We next evaluated the damage of intracellular macromolecules by PM_2.5_. The level of 8-oxoG, a hallmark of oxidative DNA damage, was measured based on 8-OHdG detection. The results indicated that PM_2.5_ promoted the generation of 8-oxoG in DNA in a time-dependent manner when used at 50 µg/mL (Fig. 1f) and in a dose-dependent manner when administered for 8 h (Fig. 1g). In addition, condensed 8-oxoG staining was observed in PM_2.5_-treated cells, whereas 1 mM NAC reduced it (Fig. 1h). The Comet assay assessing DNA breakage indicated that PM_2.5_ increased the presence of cellular DNA tails by 30% compared to control; however, NAC pre-treatment reduced it to 12% (Fig. 1i). Furthermore, fluorescence intensity of DPPP oxide, an indicator of lipid peroxidation, was enhanced in PM_2.5_-incubated cells but significantly reduced by NAC pre-treatment (Fig. 1j). Similarly, the level of protein carbonylation, a biomarker of oxidative stress-induced protein damage, was increased in PM_2.5_-treated cells, whereas NAC could prevent PM_2.5_-induced carbonyl formation (Fig. 1k). To validate these results in vivo, we used a zebrafish model. As shown in Fig. 1l, PM_2.5_ treatment also upregulated ROS production in zebrafish, which was attenuated by NAC. Finally, the results were confirmed in a mouse model, which showed that PM_2.5_ treatment induced DNA damage as indicated by the expression of phosphorylated histone H2A.X (Fig. 1m), and stimulated lipid peroxidation (Fig. 1n) and protein carbonylation (Fig. 1o) in mouse skin. Overall, these findings show that PM_2.5_ induced oxidative stress by enhancing ROS production, which resulted in the damage of cellular components.

### PM_2.5_-induced oxidative damage caused ER stress

**Fig. 2:**
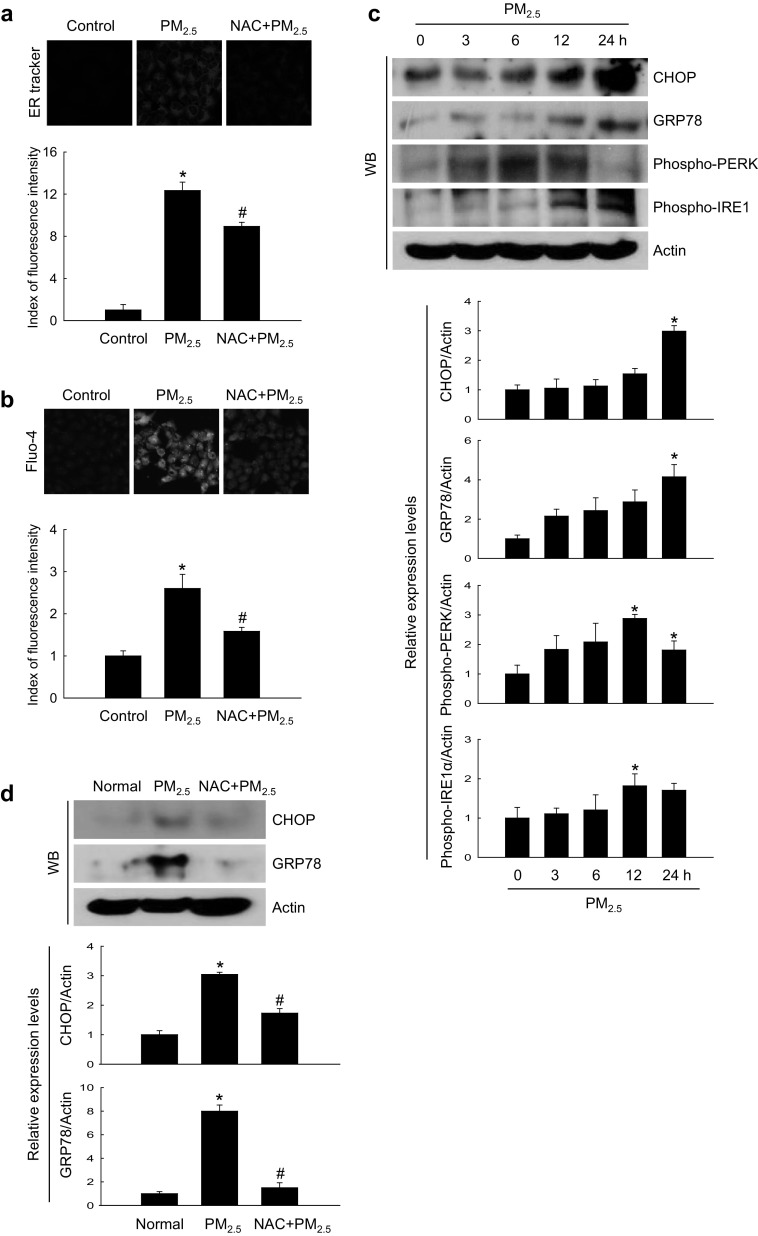
PM_2.5_ induces ER stress via ROS generation. **a, b** Cells were pre-treated or not with NAC (1 mM) for 1 h and then with PM_2.5_ (50 µg/mL) for 24 h and analyzed by confocal microscopy for ER stress using a ER-Tracker Blue-White DPX staining and for intracellular Ca^2+^ levels using b Fluo-4-AM staining. Representative confocal images are shown. Lysates of **c** cells and **d** mouse skin tissue were analyzed for the expression of CHOP, GRP78, phospho-PERK, and phospho-IRE1 by western blotting. Actin was used as loading control. *p < 0.05 compared to control groups and ^#^*p* < 0.05 compared to PM_2.5_-treated groups

We next investigated whether PM_2.5_ oxidative effects resulted in ER stress. PM_2.5_-treated cells were stained bright blue by the ER‑Tracker Blue-White DPX, indicating the induction of ER stress which was attenuated by NAC (Fig. 2a), suggesting that PM_2.5_ promoted ER stress through ROS generation. ER is a major intracellular Ca^2+^ reservoir, and disruption of Ca^2+^ homeostasis activates ER stress (Jakobsen et al. 2008; Xu et al. 2005). Confocal microscopy analysis revealed higher intensity of Ca^2+^ fluorescence in PM_2.5_-treated cells compared with control, but the effect was reduced by NAC (Fig. 2b). ER stress promotes the expression of C/EBP homologous protein (CHOP), which mediates apoptosis (Nishitoh 2012), and of the ER chaperone and signaling regulator GRP78, which activates protein kinase R-like ER kinase (PERK) through phosphorylation. In turn, phospho-PERK causes inhibition of translation and protein synthesis observed after ER stress (Bertolotti et al. 2000). As shown in Fig. 2c, PM_2.5_ induced the expression of ER stress-related proteins, including CHOP, GRP78, phospho-PERK, and phospho-serine/threonine-protein kinase/endoribonuclease inositol-requiring enzyme 1 (IRE1) in a time-dependent manner. To confirm these results, we examined the induction of ER stress markers in mice treated with PM_2.5_ and found that the expression of GRP78 and CHOP was significantly increased in PM_2.5_-treated skin compared with control; however, NAC reversed the effect (Fig. 2d). These observations were consistent with those in vitro, suggesting that PM_2.5_-induced ER stress may be associated with oxidative stress.

### PM_2.5_-induced oxidative stress promoted mitochondrial damage

**Fig. 3:**
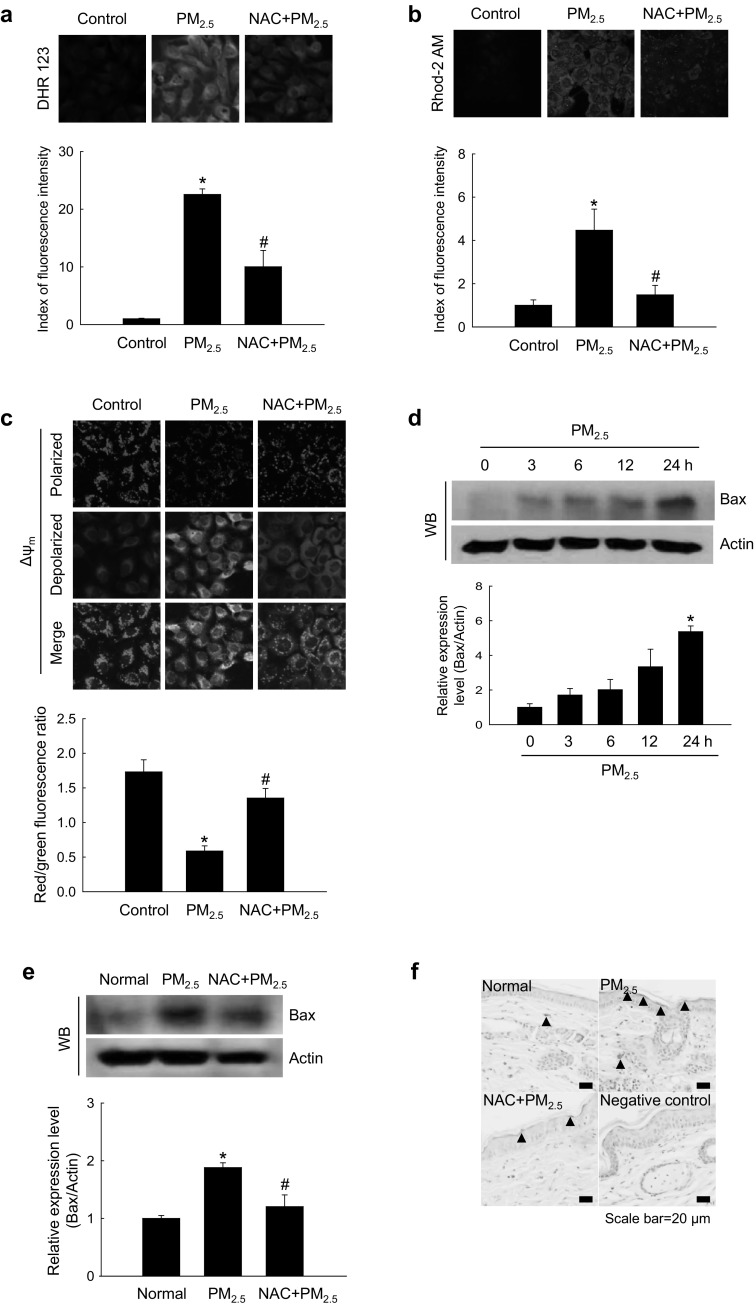
PM_2.5_ induces mitochondrial damage via ROS generation. Cells were pre-treated or not with NAC (1 mM) for 1 h and then with PM_2.5_ (50 µg/mL) for 24 h and analyzed by confocal microscopy to assess **a** mitochondrial ROS (DHR123 staining), **b** mitochondrial Ca^2+^ levels (Rhod-2 AM staining), and **c** Δ_ψm_ (JC-1 staining). Lysates of **d** cells and **e** mouse skin tissue were analyzed for the expression of Bax protein by western blotting. **f** Mouse skin tissue was analyzed for Bax expression by immunohistochemistry. Nuclei were stained with hematoxylin; arrows indicate Bax. *p < 0.05 compared to control groups and ^#^*p* < 0.05 compared to PM_2.5_-treated groups

Confocal microscopy analysis showed that, in cultured human keratinocytes, ROS generation and Ca^2+^ overload in mitochondria were enhanced by PM_2.5_ treatment but reduced by NAC (Fig. 3a, b). Mitochondrial membrane permeability is associated with apoptosis through the release of cytochrome *c* and caspase activation (Chaudhary et al. 2016). A membrane-permeant dye JC-1 is widely used in apoptosis studies to monitor the status of mitochondria where JC-1 accumulates in a potential-dependent manner as indicated by a fluorescence emission shift from green (~ 529 nm) to red (~ 590 nm). Accordingly, mitochondrial polarization (healthy state) or depolarization (damaged state) can be revealed by an increase or decrease, respectively, in the red/green fluorescence intensity ratio (Lee et al. 2017). Confocal microscopy images showed that in control cells, mitochondria exhibited strong red JC-1 fluorescence indicative of Δ_ψm_ polarization, which was reduced in PM_2.5_-treated cells where green fluorescence indicative of Δ_ψm_ depolarization was increased; however, the effect was suppressed by NAC (Fig. 3c).

It is known that proteins of the Bcl-2 family regulate apoptosis by controlling mitochondrial permeability. Therefore, we next examined whether PM_2.5_ affected the expression of Bax, a pro-apoptotic member of the Bcl-2 family. The results indicated that PM_2.5_ increased Bax levels in cultured human keratinocytes (Fig. 3d) as well as in mouse skin (Fig. 3e, f); however, NAC pre-treatment prevented PM_2.5_-induced upregulation of Bax expression (Fig. 3e, f). Cumulatively, these data indicate that PM_2.5_ increased oxidative stress in mitochondria by stimulating ROS production, which resulted in mitochondrial damage.

### PM_2.5_-induced oxidative stress caused autophagy

**Fig. 4:**
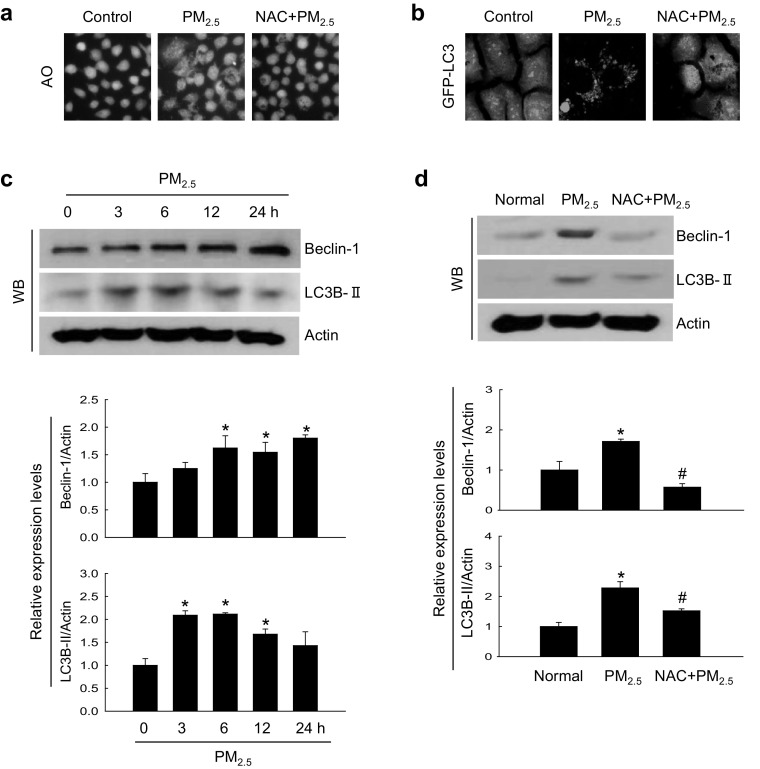
PM_2.5_ induces autophagy via ROS generation. Cells were pre-treated or not with NAC (1 mM) for 1 h, treated with PM_2.5_ (50 µg/mL) for 24 h. **a** Cells were stained with acridine orange and analyzed for autophagy by fluorescence microscopy. **b** Cells were transfected with the GFP-LC3 expression construct and observed under a fluorescence microscope. Lysates extracted from **c** cells and **d** mouse skin tissue were analyzed for the expression of beclin-1 and LC3B-II proteins by western blotting; actin was used as loading control. *p < 0.05 compared to control groups and ^#^*p* < 0.05 compared to PM_2.5_-treated groups. (Color figure online)

We next determined whether PM_2.5_-induced oxidative stress could promote autophagy. In cultured keratinocytes, PM_2.5_ triggered accumulation of intracellular vacuoles indicative of autophagy, as evidenced by staining with a lysosome marker dye acridine orange (Fig. 4a). The two distinct steps of autophagy, autophagosome formation and autolysosome formation, can be discerned by the presence of LC3-phospholipid conjugates (Tanida et al. 2005). PM_2.5_-treated GFP-LC3-transfected cells had increased levels of GFP-LC3-positive puncta (Fig. 4b). In addition, PM_2.5_ upregulated the expression of beclin-1, the protein initiating autophagosome formation during autophagy, and LC3B-II, the processed form of LC3, in a time-dependent manner (Fig. 4c). These in vitro results were confirmed in PM_2.5_-treated mouse skin (Fig. 4d). However, the effects of PM_2.5_ both in cultured cell and animals were reversed by NAC (Fig. 4a, b, d), suggesting that PM_2.5_ increased autophagy through oxidative stress.

### PM_2.5_-induced oxidative stress promoted apoptotic cell death

**Fig. 5:**
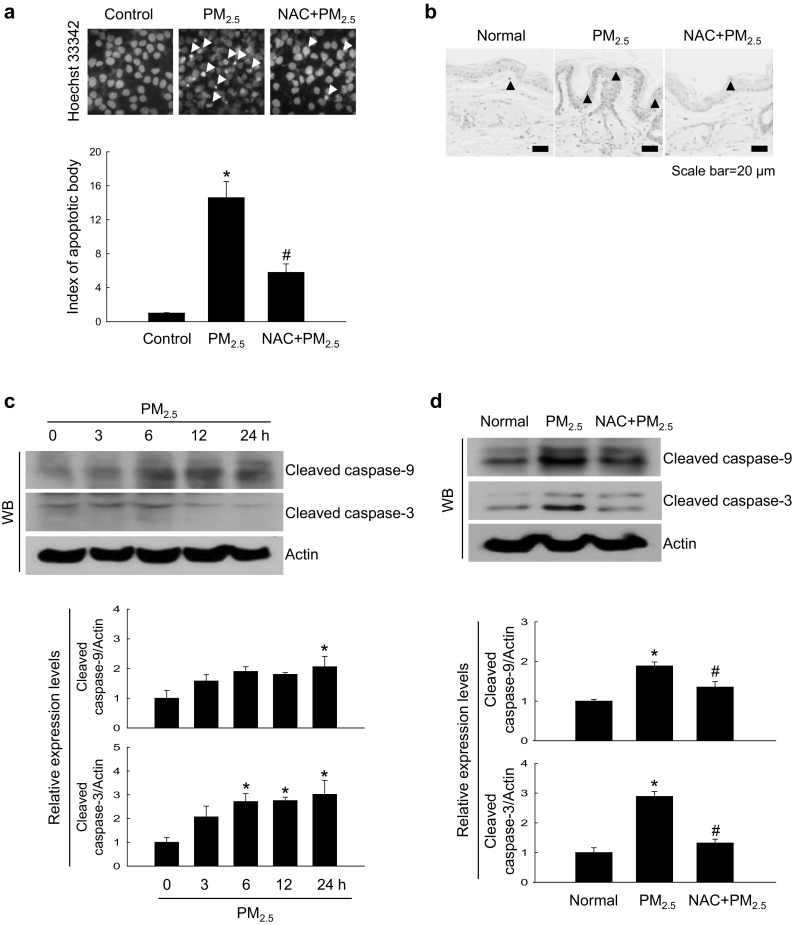
PM_2.5_ induces apoptosis via ROS generation. **a** Cells were pre-treated or not with NAC (1 mM) for 1 h, treated with PM_2.5_ (50 µg/mL) for 24 h, and analyzed for apoptotic body formation after Hoechst 33342 staining; apoptotic bodies are indicated by arrows. **b** Mouse skin treated with NAC and PM_2.5_ (100 µg/mL) for 7 days was analyzed for apoptosis by TUNEL staining; TUNEL-positive cells are indicated by arrows. Lysates extracted from **c** cells and **d** mouse skin tissue were analyzed for the expression of caspase-3 and caspase-9 by western blotting. *p < 0.05 compared to control groups and ^#^*p* < 0.05 compared to PM_2.5_-treated groups

PM_2.5_ induced apoptosis both in cultured cells and mouse skin tissues, as shown by the formation of apoptotic bodies and DNA fragmentation revealed by Hoechst 33342 staining and TUNEL assay, respectively; however, NAC pre-treatment diminished the effects (Fig. 5a, b). Another evidence that PM_2.5_ promoted apoptosis was time-dependent increase in the expression of cleaved caspase-9 and caspase-3 (Fig. 5c), which indicated caspase activation in response to mitochondrial membrane disruption. Similar results were obtained for the mouse skin, where cleaved forms of caspase-3 and caspase-9 were upregulated in response to PM_2.5_ treatment; however, the effect was attenuated by NAC (Fig. 5d), suggesting that PM_2.5_ induced apoptosis via oxidative stress.

### PM_2.5_ internalization damaged the ultrastructure of mouse skin tissue

**Fig. 6:**
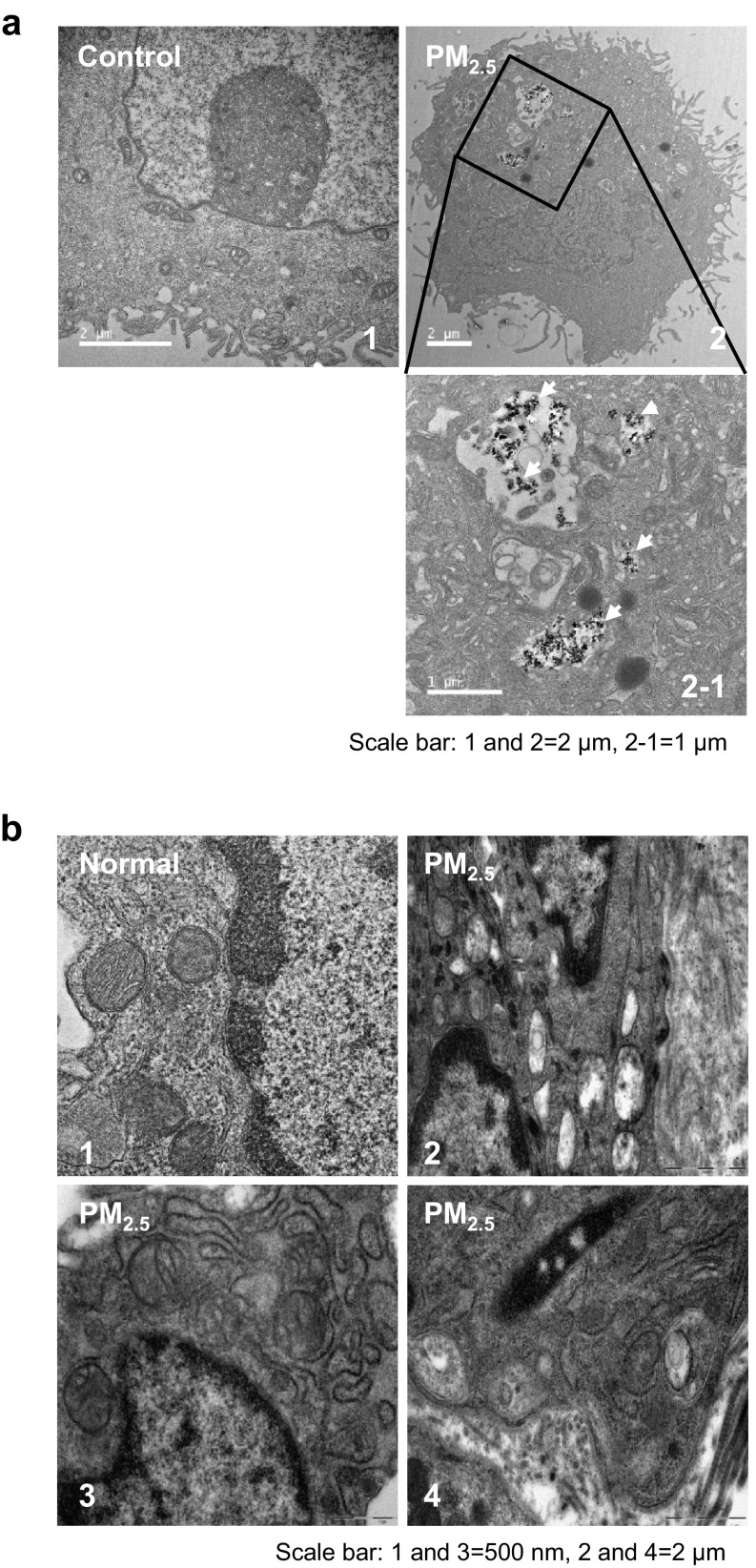
TEM analysis of HaCaT cell and mouse skin after PM_2.5_ treatment. **a** Cells were treated with 50 µg/mL PM_2.5_ for 24 h and analyzed for PM_2.5_ internalization (white arrow); **1** control, **2** internalized PM_2.5_. Scale bars: **1** and **2**, 2 µm; **2** − **1**, 1 µm. **b** Mouse skin treated or not with 100 µg/mL PM_2.5_ for 7 days. Compared to **1** normal untreated skin tissue, PM_2.5_-treated skin showed swelling of **2** mitochondria and **3** ER, and the presence of **4** autophagosomes. Scale bars, **1** and **3**, 500 nm; **2** and **4**, 2 µm

TEM analysis revealed PM_2.5_ internalization in HaCaT cells after 24 h of exposure to 50 µg/mL PM_2.5_ (Fig. 6a-2). In addition, to evaluate organelle ultrastructure in skin cells following PM_2.5_ exposure, we performed TEM analysis of mouse skin tissue after treatment with 100 µg/mL PM_2.5_. Compared to normal mice (Fig. 6b-1), skin tissue of PM_2.5_-treated mice showed increased swelling of mitochondria (Fig. 6b-2) and ER (Fig. 6b-3), and autophagosome formation (Fig. 6b-4), indicating that PM_2.5_ disrupted intracellular network in the skin.

## Discussion

According to the Air Korea site of Korea Environment Corporation (2015, 2016, 2017) of the National Environmental Research Institute of the Republic of Korea, the average concentrations of PM_2.5_ in the air of seven major Korean cities from January to March were 31, 28, and 29 µg/m^3^ in 2015, 2016, and 2017, respectively, exceeding the national environmental standard of 25 µg/m^3^.

Skin keratinocytes present the first barrier for environmental pollutants, and it was shown that PM exposure could upregulate pro-inflammatory mediators and AhR expression, leading to increased ROS generation in keratinocytes (Choi et al. 2011). Wei et al. (2017) demonstrated that organic extracts containing PAHs with PM_2.5_ induced stronger oxidative stress compared to those without PM_2.5_. It was also shown that PM_2.5_ may penetrate the skin and have harmful effects on viable skin cells, including keratinocytes (Krutmann et al. 2014; Li et al. 2017). A recent study demonstrated that PM_2.5_ could increase ROS production and inhibit the intracellular antioxidant system, which resulted in morphological changes and decreased viability of keratinocytes (Hu et al. 2017). Therefore, in the current study, we investigated the effects of oxidative stress induced by PM_2.5_ on keratinocytes in vitro and in vivo. Our data indicate that PM_2.5_ treatment promoted ROS generation (Fig. 1a, c) and caused structural damage, including DNA oxidation, lipid peroxidation, and protein carbonylation (Fig. 1f–k). Recent studies have shown that ER stress is associated with oxidative stress and that ROS may act as messengers between these processes (Cao and Kaufman 2014; Laing et al. 2010). Excessive ER Ca^2+^ release and mitochondrial Ca^2+^ overload further amplify oxidative stress (Ly et al. 2017). Therefore, we hypothesized that ROS overproduction induced by PM_2.5_ affected the ER which plays an important role in cellular quality control and sensitivity to oxidative stress. Abnormal ER stress is associated with autophagy-induced protein degradation and activation of cytotoxic processes such as apoptosis (Schrock et al. 2013), which may be a key mechanism underlying PM_2.5_ toxicity. GRP78 is a major ER chaperone critical for protein quality control in the ER and activation of ER transmembrane signaling molecules (Wang et al. 2009). GRP78 interacts with misfolded proteins and promotes their refolding, thereby playing an important role in regulating three ER transmembrane proteins: PERK, IRE-1α, and activating transcription factor 6 (ATF6) (Mei et al. 2013). Our data show that PM_2.5_ could induce IRE-1 phosphorylation, upregulate GRP78 and CHOP expression, and activate the ER stress pathway in human keratinocytes (Fig. 2c, d). Furthermore, ER stress is known to be strongly associated with the disruption of cellular Ca^2+^ homeostasis, and our results revealed that PM_2.5_-induced ER stress increased intracellular Ca^2+^ levels, which was inhibited by NAC (Fig. 2b).

Mitochondria are considered the main source of intracellular ROS and mitochondrial dysfunction plays an important role in the pathogenesis and/or progression of various diseases. Our results demonstrate that PM_2.5_ induced structural alterations of mitochondria, including swelling, which can deregulate the functional activity of the mitochondrial respiratory chain and the production of ROS, and lead to mitochondrial damage, suggesting that PM_2.5_ exposure promotes oxidative stress through destruction of mitochondria.

Autophagy is a regulated process of degradation and recycling of dysfunctional organelles and proteins, which are sequestered into autophagosomes that subsequently fuse with lysosomes where the cargo is degraded by lysosomal hydrolases (Ryter et al. 2013); however, excessive autophagy can directly cause cell death (Fulda and Kögel 2015). It has been reported that PM_2.5_-induced oxidative stress could trigger autophagy in various cell types (Deng et al. 2013, Su et al. 2017; Zhou et al. 2017). Consistent with these findings, we observed stimulation of autophagy in PM_2.5_-treated HaCaT keratinocytes in vitro and mouse keratinocytes in vivo.

**Fig. 7:**
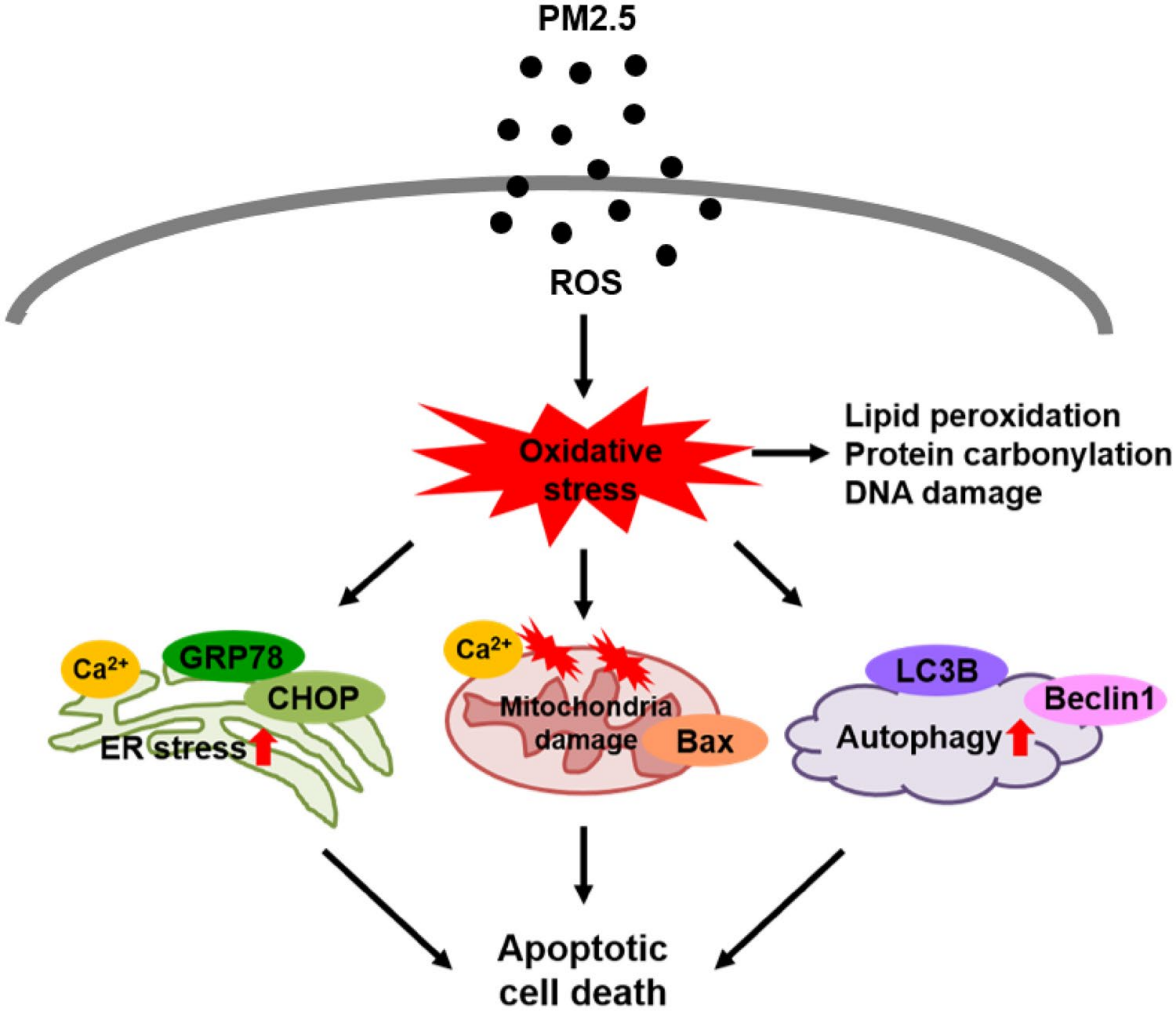
PM_2.5_ causes skin injury by increasing apoptosis through oxidative stress and destruction of cellular organelles. PM_2.5_-induced ROS generation promotes ER stress, mitochondrial dysfunction, and autophagy, leading to apoptotic cell death and skin damage

In conclusion, our study shows that PM_2.5_ causes skin damage through induction of oxidative stress, which results in the destruction of complex macromolecules and cellular organelles, including the ER, mitochondria, and lysosomes, and promotes apoptotic cell death (Fig. 7). Thus, our results contribute to understanding of the mechanisms underlying PM_2.5_-induced adverse effects on the skin.
